# Overexpression of NNMT in Glioma Aggravates Tumor Cell Progression: An Emerging Therapeutic Target

**DOI:** 10.3390/cancers14143538

**Published:** 2022-07-21

**Authors:** Wei Sun, Yongxiang Zou, Zheng Cai, Jinxiang Huang, Xinjie Hong, Qiang Liang, Weilin Jin

**Affiliations:** 1 Department of Neurosurgery, Shanghai Institute of Neurosurgery, Changzheng Hospital, Second Military Medical University, Shanghai 200003, China; caizheng180@smmu.edu.cn (Z.C.); hjx5732@126.com (J.H.); cnhxjie@smmu.edu.cn (X.H.); liangqiang8@126.com (Q.L.); 2 Neurosurgery Department, The Air Force Hospital of Northern Theater PLA, Shenyang 110041, China; bigxiang1987@hotmail.com; 3 Department of Pharmacology, Changhai Hospital, Second Military Medical University, Shanghai 200433, China; 4 Institute of Cancer Neuroscience, Medical Frontier Innovation Research Center, The First Hospital of Lanzhou University, The First Clinical Medical College of Lanzhou University, Lanzhou 730000, China

**Keywords:** nicotinamide *N*-methyltransferase, glioma, GAP43, NAD/NADH ratio, SIRT1

## Abstract

**Simple Summary:**

Glioma is one of the most common intracranial malignancies and is incurable due to strong aggressiveness and resistance to radiotherapy and chemotherapy. The lack of effective therapeutic targets is a major problem in current treatment. In the present study, we found that nicotinamide *N*-methyltransferase (NNMT) is a key factor influencing the occurrence and development of glioma. High NNMT expression in glioma is a predictor of short overall survival and poor patient outcome. NNMT knockdown reduced the volume of mice xenograft glioma and the viability of glioma cells. Additionally, overexpression of NNMT epigenetically silenced GAP43 through DNA methylation, histone methylation, and deacetylation modification processes. GAP43 can inhibit the formation of microtubules in tumor and intertumor cell network connections and induce apoptosis through the SIRT1 signaling pathway. Therefore, NNMT could be a potential candidate for the clinical diagnosis and treatment of glioma.

**Abstract:**

Purpose: Increasing evidence has revealed that nicotinamide *N*-methyltransferase (NNMT) is a key factor influencing the prognosis of tumors. The present study aimed to investigate the role of NNMT in glioma and to elucidate the associated functional mechanisms. Methods: Clinical samples were analyzed by immunohistochemical staining and Western blotting to evaluate NNMT expression in glioma and normal brain tissues. The correlation between NNMT expression and glioma was analyzed using the Cancer Genome Atlas (TCGA) database. Additionally, NNMT was knocked down in two types of glioma cells, U87 and U251, to evaluate the invasive ability of these cells. Quantitative real-time polymerase chain reaction (qRT-PCR) was used to validate NNMT knockdown in the cells. Furthermore, ELISA was used to determine the balance between nicotinamide adenine dinucleotide and nicotinamide adenine dinucleotide hydrogen (NAD/NADH ratio), which verified the altered methylation patterns in the cells. The glioma xenograft mouse models were used to verify the regulatory role of NNMT, GAP43, and SIRT1. Results: Analysis based on our clinical glioma samples and TCGA database revealed that overexpression of NNMT was associated with poor prognosis of patients. Knockdown of NNMT reduced the invasive ability of glioma cells, and downregulation of its downstream protein GAP43 occurred due to altered cellular methylation caused by NNMT overexpression. Gene Set Enrichment Analysis confirmed that NNMT modulated the NAD-related signaling pathway and showed a negative association between NNMT and SIRT1. Moreover, the regulatory roles of NNMT, GAP43, and SIRT1 were confirmed in glioma xenograft mouse models. Conclusion: Overexpression of NNMT causes abnormal DNA methylation through regulation of the NAD/NADH ratio, which in turn leads to the downregulation of GAP43 and SIRT1, eventually altering the biological behavior of tumor cells.

## 1. Introduction

Glioma is a tumor of neuroepithelial origin and is the most common type of cancer in the central nervous system. Glioma has the highest incidence of brain tumors, which are characterized by its tendency to recur [1,2]. Typically, headaches, vomiting, hemiparesis, and seizures may be clinical manifestations in patients with glioma, where the glioma invades peripheral neurons and nerve fibers. The proliferation of gliomas in the brain can lead directly to brain dysfunction and neurological disease. To make matters worse, gliomas can spread by blood and lymphatic routes, making them highly aggressive, which is why the prognosis for patients is generally poor [3,4]. According to the World Health Organization (WHO), glioma is classified into four grades: grade I, pilocytic astrocytoma; grade II, diffuse astrocytoma; grade III, anaplastic astrocytoma; and grade IV, glioblastoma (GBM) [5,6]. Both grade I and II gliomas are low-grade gliomas (LGGs), and grade III and IV gliomas are high-grade gliomas (HGGs). HGG is the most common type of malignant intracranial tumor with poor prognosis. There is no mature scheme for the palliative treatment of HGG, nor is there standard treatment for recurrent and drug-resistant HGG [7]. Patients with glioma are treated in a similar manner to other tumors. The primary treatment strategies used to inhibit tumor growth are surgical resection, radiotherapy, and chemotherapy. The current prognosis and survival rate for patients with HGG remain poor, with a 5-year survival rate of less than 5% [8,9,10].

Nicotinamide *N*-methyltransferase (NNMT) is a methyltransferase that substantially influences the global methylation in cells [11]. The physiological role of NNMT is to utilize S-adenosyl-L-methionine (SAM) to transfer methyl to nicotinamide (NAM), producing 1-methylnicotinamide (1MNA) and releasing S-adenosyl-L-homocysteine (SAH) [12,13]. Currently, the analysis of proteomic data has revealed that NNMT is expressed in various cancer types, including breast cancer [14], bladder cancer [15], gastric cancer [12,16], liver cancer [12], colorectal cancer [17], ovarian cancer [18], oral squamous cell carcinoma [19], and skin cancer [20].

Tumor development is usually accompanied by the formation of a tumor bed, massive changes in the surrounding connective tissues and matrix, and the formation of a microenvironment suitable for the survival of tumor cells. A previous study reported in *Nature* [21] revealed that the stromal cells surrounding cancer cells made tumors to be more malignant, aggressive, and invasive. A systematic examination of tumors and the surrounding fibroblasts has revealed a metabolic enzyme, NNMT, which is highly expressed in the matrix surrounding metastatic cancer cells. NNMT is a key regulatory molecule in the maintenance of fibroblast phenotype, and it considerably regulates most genes in the tumor stroma, thereby transforming normal fibroblasts into cancer-associated fibroblasts that support and accelerate tumor growth. Overexpression of NNMT in the stroma promotes the progression and metastasis of ovarian cancer and interferes with the prognosis of patients. Several studies have demonstrated that NNMT could also be involved in the response to chemotherapeutics. Overexpression of NNMT changed the expression and activity of Sirt1 in breast cancer tissues, which in turn significantly inhibited apoptosis induced by adriamycin and paclitaxel and increased tumor resistance [14], while downregulation of NNMT was associated with increased sensitivity of melanoma cells to dacarbazine therapies [22]. The expression of NNMT was also found to be associated with the proliferation of colorectal cancer and its sensitivity to 5-fluorouracil [23]. In biological systems, NNMT can compete with leucine carboxyl methyltransferase 1 (LCMT1) for methylation. Inhibition of NNMT expression relatively increased the potential for methylation of LCMT1 and led to a reduction in the risk of tumor formation. In addition, inhibition of NNMT preserves the radiosensitizing effect of nicotinamide and enhances radiosensitivity [24,25]. Although there is some controversy regarding NNMT as a precancerous and prognostic marker, some studies have demonstrated the benefits of NNMT inhibition in both tumor invasion and aggression [26,27].

## 2. Materials and Methods

### 2.1. Clinical Samples

Patients with glioma who underwent surgical treatment between 2016 and 2019 were recruited for the present study. A total of 59 gliomas were collected from the patients and pathologically confirmed. Moreover, 10 nontumor tissue specimens were obtained from patients with severe brain trauma who required decompression treatment. The gliomas were graded according to the pathological diagnostic standards of the WHO. No patient had received radiotherapy or chemotherapy prior to tumor resection. Samples were divided and either frozen in liquid nitrogen and stored at −80 °C or stored in RNA later (Ambion, Austin, TX, USA) at −20 °C. Ethical approval for the study was sought from the Independent Ethics Committee of Second Military Medical University. Verbal and written informed consents were obtained from all patients or their guardians according to the guidelines of the Ethics Committee.

### 2.2. Immunohistochemical Analysis

Glioma tissue sections (5 µm) were routinely dewaxed and hydrated. The sections were treated with 0.3% hydrogen peroxide after antigen repair reduced endogenous peroxidase activity and incubated with one drop of blocking solution for 5 min at room temperature. Afterward, one drop of the primary antibody working solution was added to the sections and incubated for 1–2 h at 37 °C. Secondary antibodies of the corresponding species were added and incubated for 1 h. Finally, the sections were counterstained with hematoxylin and eosin (H&E) stain to determine the location of the nuclei. The sections were washed with phosphate-buffered saline (PBS) after each step.

### 2.3. Western Blot Assay

Proteins from glioma tissue samples were extracted using radioimmunoprecipitation assay buffer and centrifuged to obtain a supernatant consisting of protein extracts. The concentrations of the extracted proteins were determined using the BCA protein assay kit (Beyotime Biotechnology, Shanghai, China), and equal volumes of sample concentrations (20 μg/20 μL) were prepared. Proteins were separated using 10% sodium dodecyl sulfate polyacrylamide gel electrophoresis (SDS-PAGE) and transferred onto polyvinylidene fluoride (PVDF) membranes (Merck Millipore, Burlington, MA, USA). Then, PVDF membranes were transferred to the blocking solution containing 5% BSA for a 2 h reaction at room temperature. The PVDF membranes were coincubated with primary antibodies at 4 °C overnight after washing. Subsequently, the PVDF membranes were incubated with horseradish peroxidase-conjugated secondary antibodies for 1.5 h at room temperature. Protein concentrations were detected using enhanced chemiluminescence (Pierce Biotechnology, Rockford, IL, USA), and the gel images were analyzed using a gel imaging system. The results of the final Western blot were quantified with GAPDH as the control for normalization. The manufacturer of the antibodies used and their dilutions are shown in Table 1.

### 2.4. Quantitative Real-Time Polymerase Chain Reaction

RNA from tumor samples was extracted using Trizol reagent (Invitrogen, Carlsbad, CA, USA) according to the manufacturer’s protocol. The reverse transcription reaction was performed using the SYBR^®^ Premix Ex Taq™ (Takara, RR420A) RT-PCR kit. The primers were designed and synthesized, and quantitative real-time polymerase chain reaction (qRT-PCR) was performed according to the manufacturer’s instructions. GAPDH was used as an internal reference for mRNA. The relative quantitative method (2^−ΔΔCT^ method) was used to calculate the relative expression of mRNA. The primer sequences are shown in Table 2.

### 2.5. Cell Cultures

Glioma cell lines (U87 and U251) were cultured in Dulbecco’s modified eagle medium (Gibco BRL, Grand Island, NY, USA) supplemented with 10% fetal bovine serum (FBS) and 1% penicillin/streptomycin. The cells were cultured in a humidified environment of 95% air and 5% carbon dioxide (CO_2_) at 37 °C. The culture medium was replaced once every 1–2 days.

### 2.6. Cell Viability Assay

The construction of lentiviral vectors (hU6-MCS-pUb-EGFP-IRES-puromycin) was ordered from GeneChem Co., Ltd. (Shanghai, China). The target sequence for NNMT shRNA 1# was 5′-GCTCAAGAGCAGCTACTACAT-3′, and for NNMT shRNA 2# was 5′-TGCAGAAAGCCAGATTCTTAA-3′. U251MG and U87MG cells cultured in 6-well corning Costar plates for 24 h were transfected with lentivirus. Eight hours later, the culture medium was replaced with a fresh medium. After 48 h, the transfected cells were subcultured into 25 cm^2^ corning flasks in a ratio of 1:10 with 1.5 μg/mL puromycin (Thermo Fisher Scientific, Inc., Waltham, MA, USA). The cell was continuously cultured until stably transfected cell strains were obtained. The cells were analyzed by performing the water-soluble tetrazolium-1 (WST-1) assay. In brief, the cells were seeded into a 96-well plate at a density of 5.0 × 10^4^ cells/mL (100 μL/well) and divided into the following groups: (1) normal control (NC), (2) NNMT knockdown 1 (KD1), and (3) NNMT knockdown 2 (KD2). After culturing for 1–4 days, WST-1 (2-(4-Iodophenyl)-3-(4-nitrophenyl)-5-(2,4-disulfophenyl)-2H-tetrazolium) reagent was added (10 μL/well) to each well and incubated at 37 °C for 1 h. Absorbance was measured at 450 nm using a microplate reader (Thermo Fisher Scientific, Waltham, MA, USA). Cell viability was calculated and compared with the corresponding control group [28].

### 2.7. Wound Healing Assay

Cells were equally distributed into a 6-well plate at a density of approximately 5 × 10^5^ cells/mL. Afterward, a pipette was used to make horizontal lines on the cell monolayer. Cells were washed three times with PBS to remove excess cells, followed by addition of a serum-free medium, and the cells were cultured in an incubator with 5% CO_2_ at 37 °C. Live-cell dye was added at 23 h, and the samples were photographed at 24 h.

### 2.8. Transwell Migration and Invasion Assays

Cell suspension (200 µL) containing 10^4^ cells was added to the transwell of a 24-well plate (insert size: 6.5 mm; pore size: 8 μm, Corning, New York, NY, USA). Thereafter, 500 µL of a medium containing FBS was added to the lower chamber of the 24-well plate and incubated for 24 h. The cells were subsequently stained with 0.1% crystal violet (Thermo Fisher Scientific, Waltham, MA, USA), washed twice with PBS, and incubated at room temperature for 30 min. A microscope (Olympus, Tokyo, Japan) was used for photographic recording, and the light intensity was analyzed using ImageJ software (ImageJ, NIH, Maryland, MD, USA).

### 2.9. Determination of NAD/NADH Ratio

Glioma cell samples were collected after the mice were sacrificed. Cellular proteins were determined by the BCA Protein Kit after sufficient lysis for protein concentration, and all samples tested were diluted to equal concentrations with appropriate lysate. The levels of NAD and nicotinamide adenine dinucleotide hydrogen (NADH) in glioma cells were determined by performing a quantitative colorimetric assay according to the manufacturer’s instructions. Absorbance of the reaction mixture was measured at 565 nm [29].

#### 2.9.1. Experimental Animals

Five-week-old athymic BALB/c male nude mice (nu/nu) were obtained from Shanghai SLAC Laboratory Animal Co. Ltd. (SLAC, Shanghai, China). The animals were placed in an environmentally controlled and centralized animal facility, which was maintained at 21–23 °C, light/dark cycle (12/12 h), humidity of 45–55%, and they were fed with a commercial diet and distilled water.

#### 2.9.2. Hematoxylin and Eosin Staining

H&E staining was performed according to a previously described method [30]. Briefly, after dewaxing and hydration, a 5 μm longitudinal section was stained with hematoxylin solution for 7 min, soaked in 1% hydrochloric acid in ethanol for 3 s, followed by soaking in 2% ammonia for 10 s. The sections were stained with eosin solution for 5 min and then dehydrated with graded ethanol and xylene. Finally, a microscope was used to take photographs.

#### 2.9.3. Statistical Analyses

Statistical analyses were carried out using R version 3.3.4 (www.r-project.org, accessed on 11 July 2021). Data were expressed as means ± standard error of the mean (SEM). Significant differences in mean values among groups were determined using one-way analysis of variance, followed by the least significant difference post hoc tests for mean separation. Comparisons between two groups were performed using Student’s *t*-test (two-tailed). The significance level was set at *p* < 0.05.

## 3. Results

### 3.1. NNMT Expression Promotes Glioma Progression in Patients

**Figure 1:**
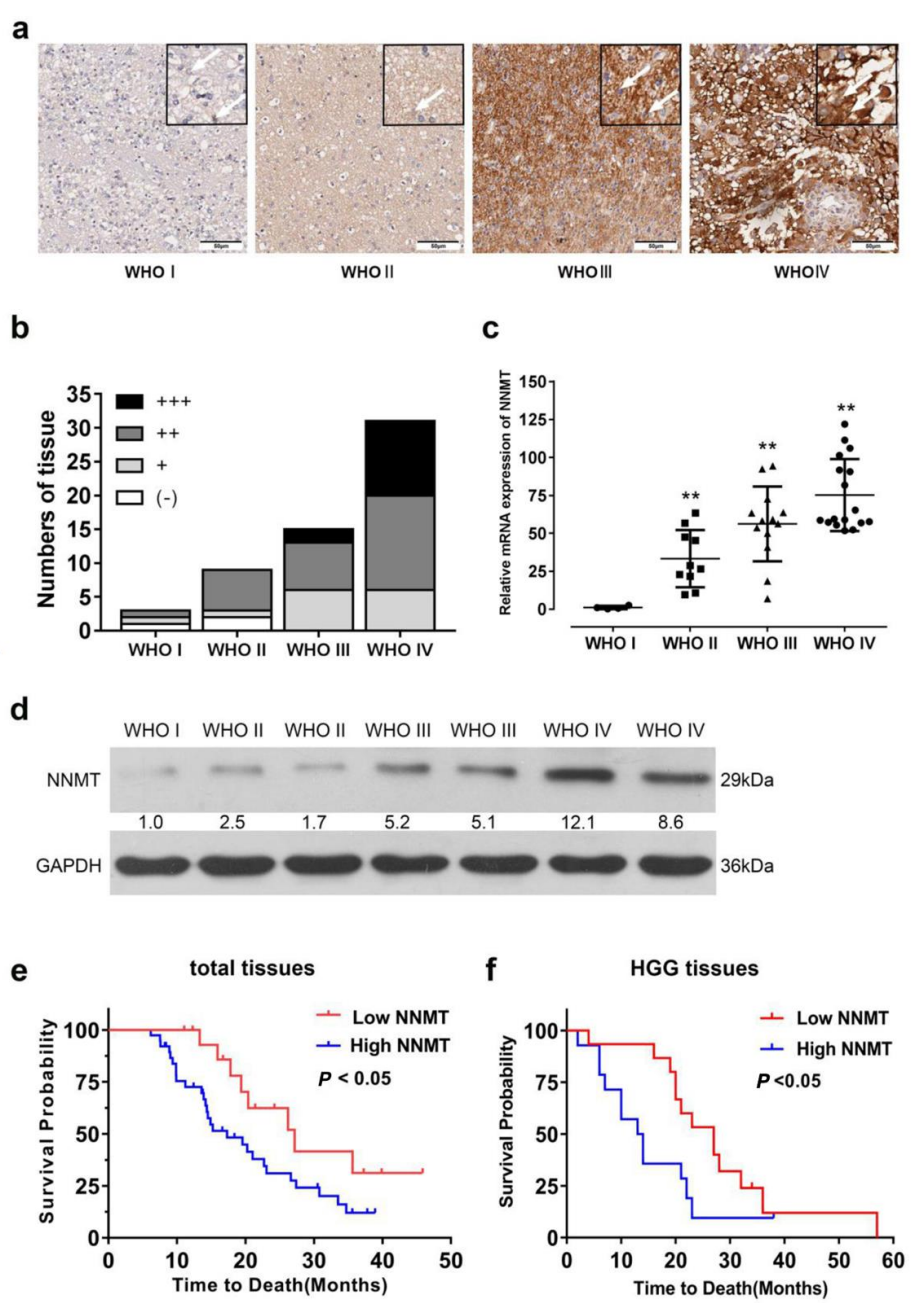
(**a**) Protein expression of NNMT based on immunohistochemical staining. (**b**) Quantitative analyses result of NNMT proteins. (**c**) mRNA expression of NNMT. ** *p* < 0.01, compared with WHO I. (**d**) Protein expression of NNMT as determined by Western blot. (**e**) Relationship between NNMT expression and survival of patients with glioma. (**f**) Relationship between NNMT expression and survival of patients with HGG. Values are expressed as means ± SEM.

Previous studies have revealed that NNMT expression increases considerably in cancer tissues [17,21,31]. Therefore, we investigated the relationship between NNMT expression and patients with glioma. Notably, the protein expression of NNMT (Figure 1a,b,d and Figure S1) increased in patients with different grades of glioma, as determined by immunohistochemical and Western blot analyses. Similar results were observed in the qRT-PCR analysis (Figure 1c). The main trend of NNMT expression levels was obtained by tissue chip; Western blot was only used for validation for this part, and consistent results were obtained (Figure 1d and Figure S1). NNMT expression levels were associated with survival probability in patients with glioma. Low expression levels of NNMT in all types of glioma tissues based on WHO classification were associated with higher survival probabilities than high expression levels of NNMT (*p* < 0.05, Figure 1e). Regarding HGG tissues, the results revealed a significant positive correlation between NNMT expression and survival probability (*p* < 0.05, Figure 1f).

### 3.2. Relationship between NNMT Expression and Glioma Based on TCGA

**Figure 2:**
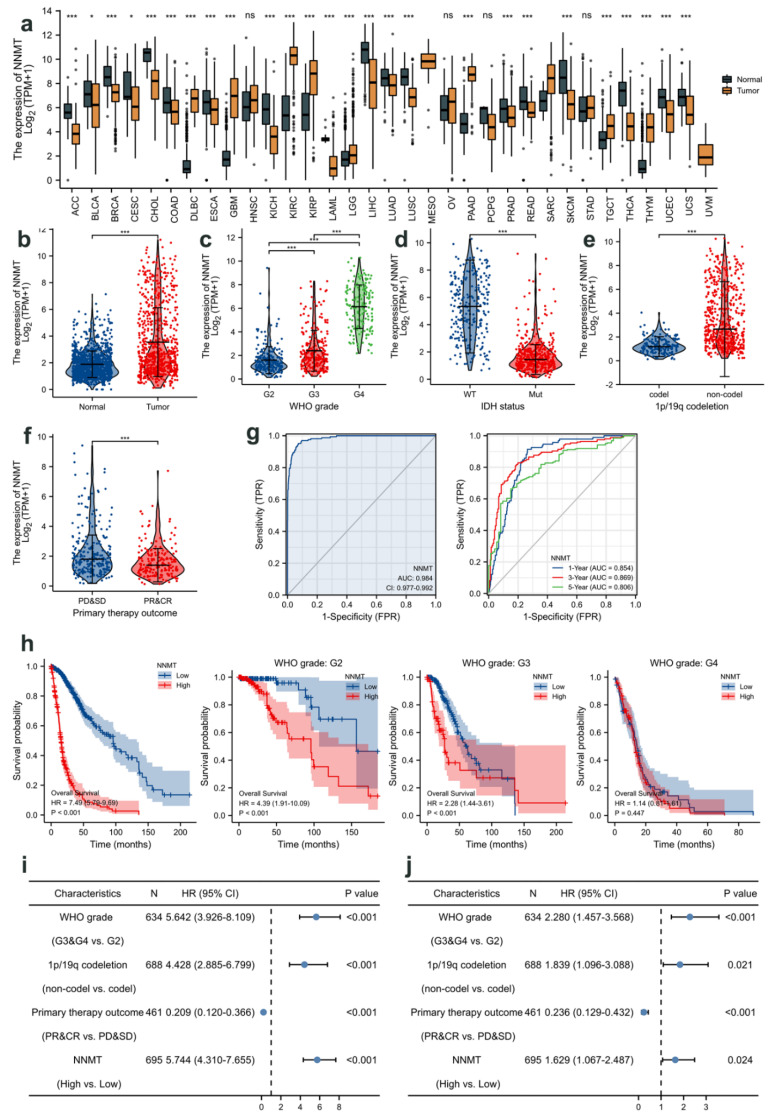
(**a**) Differential expression of NNMT in glioma and paraneoplastic tissues based on TCGA database. * *p* < 0.05, *** *p* < 0.001, compared with Normal. (**b**) Expression of NNMT in glioma of different grades based on TCGA database. (**c**) Variations in NNMT expression between patients without IDH mutation (WT) and with IDH mutation based on TCGA database. (**d**) Variations in NNMT expression between patients with 1p19q codeletion and patients without 1p19q codeletion based on TCGA database. (**e**) Variation in NNMT expression at different stages of disease progression. (**f**) Receiver operating characteristic (ROC) curve of the correlation between NNMT expression and glioma. (**g**) Relationship between survival time and NNMT expression after treatment. (**h**) Univariate Cox regression analysis of NNMT expression in the prediction of glioma grade. (**i**,**j**) Multivariate Cox regression analysis of NNMT expression in the prediction of glioma grade. Values are expressed as means ± SEM, *** *p* < 0.001.

The Cancer Genome Atlas (TCGA) database is a large, free reference database for cancer research with cancer-related histological data [32]. In this investigation, the TCGA database was used to examine the differential expression of NNMT in 33 tumor tissues, and it was found that NNMT appeared to be highly expressed in glioma (Figure 2a). The subcolumns of the brain LGG and GBM database were merged into GBMLGG for analysis using the Cancer Genome Atlas (TCGA) database. The expression of NNMT was significantly higher in tumor tissues than that in adjacent tissues (Figure 2b and Table 3). Further analysis revealed that the expression of NNMT was positively correlated with WHO grades of glioma (Figure 2c). The expression of NNMT decreased significantly in patients with glioma who had 1p19q codeletion and IDH mutation (Figure 2d,e). In addition, the expression of NNMT in patients with progressive disease and stable disease was significantly higher than that in patients with partial response and complete response (Figure 2f). Receiver operating characteristic (ROC) curve analysis revealed that the prediction ability of NNMT had high accuracy (area under the curve (AUC) = 0.984, 95% confidence interval (CI) = 0.977–0.992), and the time-dependent ROC curve analysis revealed a high accuracy at 1, 3, and 5 years (Figure 2g). Prognostic analysis revealed that the survival rate of patients with high expression of NNMT decreased significantly. Further analysis revealed that the high expression of NNMT in patients with G2 and G3 stages had a poor progressive disease and stable disease (Figure 2h). Univariate and multivariate Cox regression analyses revealed that NNMT was an independent predictor of glioma progression, and it had a significant hazard ratio to predict clinical outcomes (Figure 2i,j). The predictive value of NNMT corresponded to that of WHO grades.

### 3.3. NNMT Expression Promotes Proliferation, Migration, and Invasion of Glioma Cells

**Figure 3:**
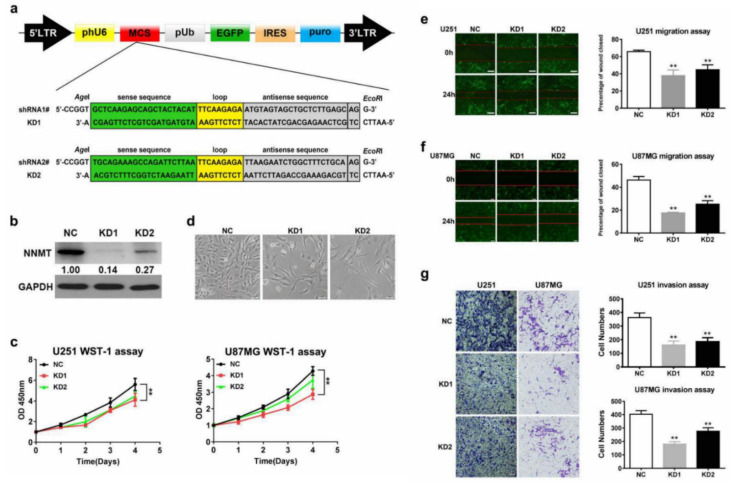
(**a**) Construction of the structural domains of two shRNAs. (**b**) Analysis results of the effect of NNMT knockdown as determined by Western blot. (**c**) Evaluation of cell proliferation using WST-1 assay. (**d**) Microscopic observation of cell growth. (**e**) Scratch assay comparing the migration of U251 glioma cells after knockdown of NNMT. (**f**) Scratch assay comparing the migration of U87 glioma cells after knockdown of NNMT. (**g**) Invasion assay comparing the variations in the invasive ability of U251 and U87 glioma cells after knockdown of NNMT. Values are expressed as means ± SEM, ** *p* < 0.01, compared with NC.

To elucidate the specific effect of NNMT expression on glioma, two shRNA-expressing vectors were constructed (Figure 3a) and proved by Western blot (Figure 3b and Figure S2). The results of the WST-1 cell proliferation experiment revealed that cell proliferation in glioma cell lines (U87 and U251) decreased in the KD1 group (Figure 3c). Moreover, cells in the KD1 group had abnormal morphology, were sparse, and were loose when compared with those of the normal group. Scratch test results of U87 and U251 glioma cells revealed that cell migration was inhibited after NNMT knockdown (Figure 3e,f). Similarly, transwell migration/invasion assay results revealed that the invasiveness of U87 and U251 cells was inhibited in the KD1 group (Figure 3g).

### 3.4. NNMT Gene Correlation Analysis

**Figure 4:**
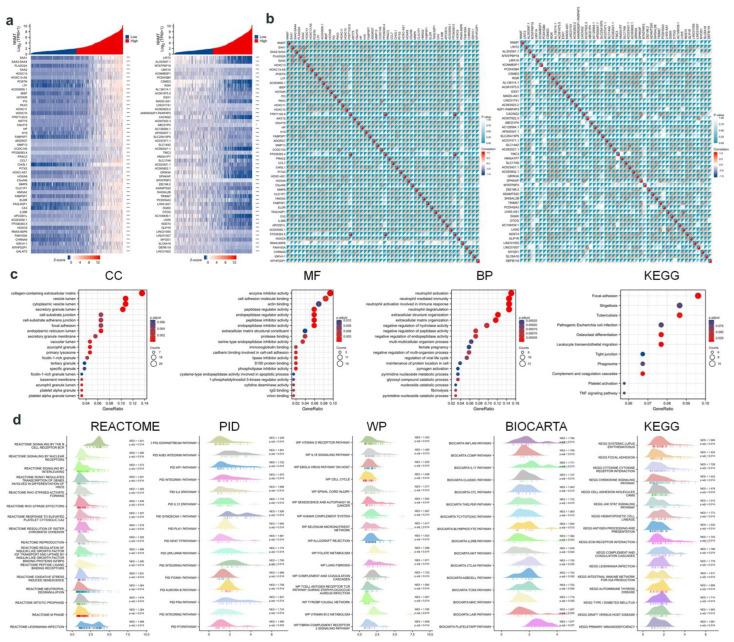
(**a**) High and low expression genes regulated by NNMT gene. (**b**) Coexpression of NNMT regulator genes. (**c**) GO (CC, MF, BP) and KEGG analyses. (**d**) GSEA analysis.

The genes regulated by NNMT genes were screened and standardized with the Z-score to classify the genes into high and low groups (Figure 4a), and the gene correlations between these two groups were statistically determined with two indicators of *p*-value and correlation (Figure 4b). Signaling pathways were enriched for NNMT-regulated related genes using the David database. The top 20 signaling pathways including cellular component (CC) analysis, biological process (BP) analysis, and molecular function (MF) in gene ontology (GO) analysis, and the top 11 KEGG signaling pathways were selected for enrichment. (Figure 4c). These related genes were enriched for signaling pathways using the Gene Set Enrichment Analysis (GSEA) database, a computational method for interpreting gene expression data based on molecular signature databases [33]. They were also analyzed in conjunction with the Reactome pathway database, pathway interaction database (PID), Wiki Pathways (WP) database, BioCarta database, and KEGG database. To better understand the biological functions and features, enrichment analysis and visual mapping of the first 16 signaling pathways were performed using R software. NES > 1.0, *p* < 0.05 was considered significantly enriched.

### 3.5. NNMT-Associated Downstream Proteins in Glioma Cells

**Figure 5:**
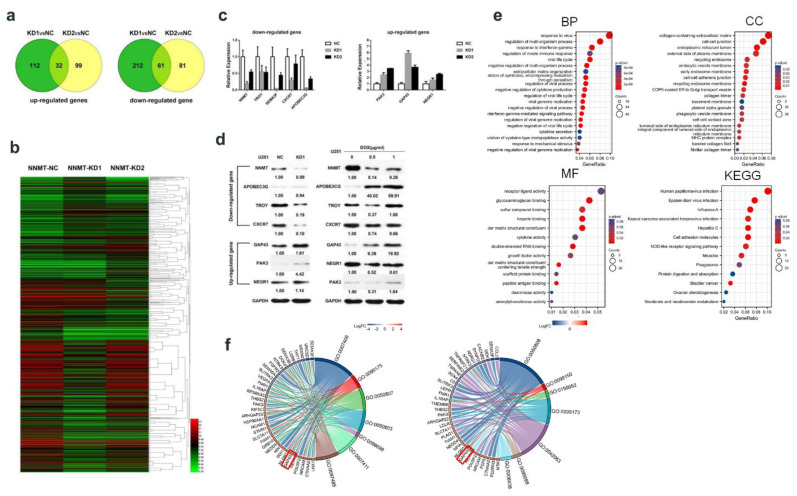
(**a**) Venn diagrams of the differential gene expression between KD1 and glioma cells, and KD2 and glioma cells. (**b**) A heat map of the differential gene expression between KD1 and glioma cells, and between KD2 and glioma cells. (**c**) qRT-PCR analysis of selected downregulated genes. (**d**) Western blot analysis of selected downregulated and upregulated genes. (**e**) GO (BP, MF, CC) and KEGG analysis. (**f**) GO analysis.

The gene expression profiles of U87 and U251 glioma cells after NNMT knockdown were analyzed and experimentally validated using qRT-PCR and Western blot. The results revealed that 32 genes were upregulated, while 61 genes were downregulated in both NNMT knockdown groups (NNMT-KD1 and NNMT-KD2 tumor cells; Figure 5a). The upregulated genes included PAK3 (p21 protein (Cdc42/Rac)-activated kinase 3), GAP43 (growth-associated protein 43), ELAVL2 (ELAVL (embryonic lethal, abnormal vision, Drosophila)-like 2), *NEGR1* (neural growth regulator 1), *STAU2* (Staufen double-stranded RNA-binding protein 2), and others. Notably, most of the genes could inhibit tumor growth. The downregulated genes included *MMP2*, *MMP7*, *RSPO3*, *SEMA3F*, *TNFRSF19*, *ANXA8*, *APOBEC3G*, *CXCR7*, and others. Most of the downregulated genes could promote tumor expansion. An in-depth data analysis was conducted on the microarray datasets, and a heat map was generated (Figure 5b). To verify whether NNMT expression affected the downstream genes, the selected genes were analyzed by qRT-PCR, and the results were consistent with the expected results (Figure 5c). The knockdown effect of NNMT in the KD1 group was significantly greater than that of the KD2 group. Therefore, the subsequent experiments involving upregulated and downregulated genes were based on the KD1 group. Since NNMT expression was inhibited in the KD1 group through doxycycline-induced shRNA inhibition, validation of the KD1 group results was performed using Western blot with two different concentrations of doxycycline-induced NNMT knockdown treatment (DOX1 and DOX 5), which revealed similar results (Figure 5d and Figure S3). For the identification of differential genes in NNMT knockdown cells, the results of BP, CC, MF, and KEGG analysis were processed using the R language to obtain bubble plots (Figure 5e). Fortunately, for the signaling pathway analyzed by GO, the GAP43 protein of our target was found to be at a high confidence level and attached to the pathway, such as “axonogenesis” (GO:0007409) and “regulation of postsynapse organization” (GO:0099175) (Figure 5f).

### 3.6. NNMT Expression Is Closely Associated with the NAD/NADH Ratio

**Figure 6:**
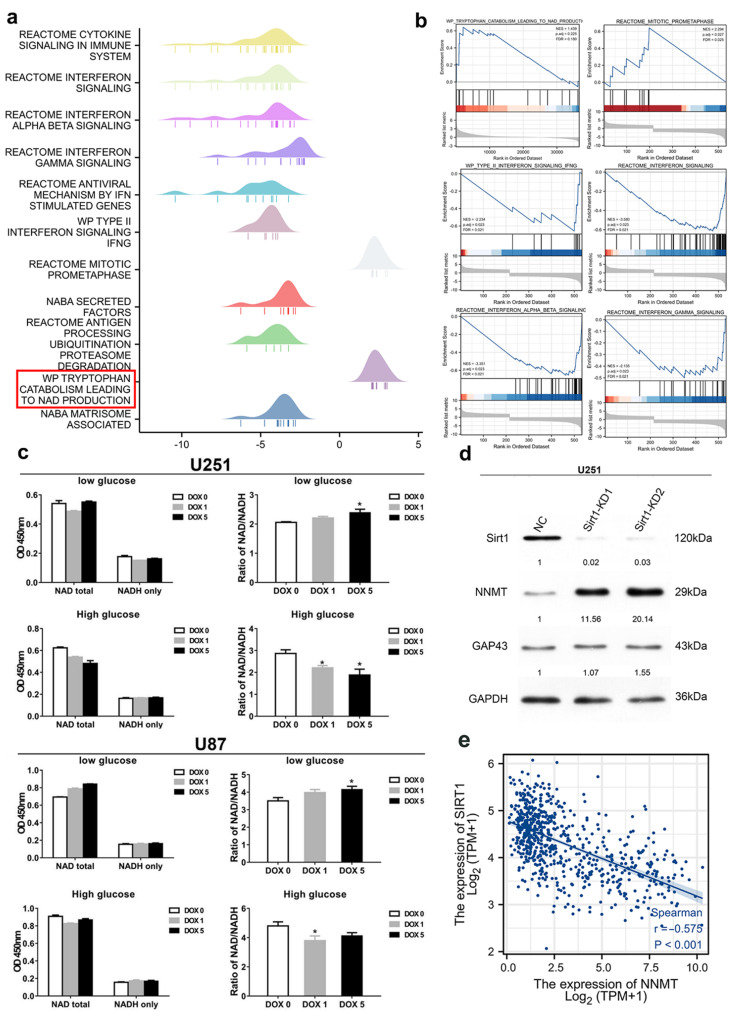
(**a**) Reactome, PID, WP, and BioCarta database analysis. (**b**) GSEA analysis. (**c**) Determination of NAD/NADH ratio in U251 andU87 glioma cells using ELISA. (**d**) Analysis results of the effect of SIRT1 knockdown by Western blot. (**e**) Correlation analysis between SIRT1 and NNMT. Values are expressed as means ± SEM, * *p* < 0.05, compared with DOX 0.

Methylation experiments revealed that the methylation of multiple genes, including the downstream gene, GAP43 was abnormal after doxycycline-induced NNMT knockdown. The Reactome pathway database, PID, WP database, and BioCarta database were used to enrich the signaling pathway for key genes such as NNMT, LHX1, CTNNA2, NEXN, and GAP43, and it was found that these key genes were mainly present in the NAD-related signaling pathway (Figure 6a). The analysis with the GSEA database revealed a strong correlation between key genes and NAD-related signaling pathways (Figure 6b). The results suggested that NNMT expression could influence the expression of GAP43 through methylation. In U251 glioma cells, knockdown of NNMT resulted in an increase in the NAD/NADH ratio in a low-glucose environment. Conversely, knockdown of NNMT decreased the NAD/NADH ratio in a high-glucose environment. Similar results were observed in U87 glioma cells. We found that NAD/NADH expression in U251 glioma cells was more susceptible to DOX challenge, which was further investigated with U251 cells (Figure 6c). A significant increase in protein expression of NNMT was found after knockdown of the SIRT1 gene in U251 cells (Figure 6d and Figure S4). The association between NNMT and SIRT1 genes was inspected from the TCGA database, and it was found that NNMT and SIRT1 presented a negative tendency of association, and the results were statistically significant (Figure 6e).

### 3.7. NNMT Knockdown Inhibits Tumor Growth by Promoting GAP43 Expression

**Figure 7:**
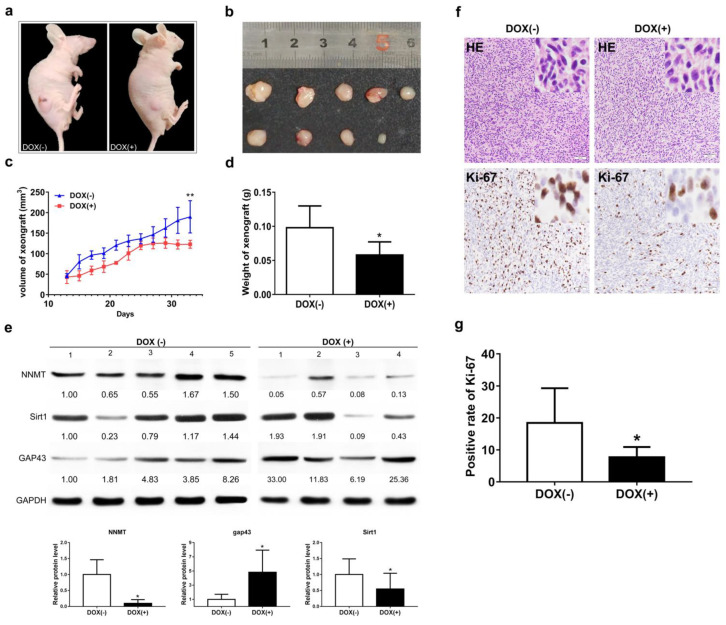
(**a**,**b**) Representative images of xenograft tumors isolated from nude mice in the different groups. (**c**) Tumor volume at different time points. (**d**) Tumor weight at different time points. (**e**) Protein expression of NNMT, SIRT1, and GAP43. (**f**) Staining of tumor sections with H&E and Ki-67 (**g**) Percentage of positive cells in Ki-67 staining. Values are expressed as means ± SEM, * *p* < 0.05, ** *p* < 0.01, compared with DOX(−).

A mouse xenograft tumor model was constructed using U251 cells because the knockdown effect of U251 induced by doxycycline was greater. The mouse model was successfully established, and tumors were observed in most mice [31]. Knockdown of NNMT exerted an inhibitory effect on the growth of solid tumors in nude mice (Figure 7a,b). The volumes and weights of solid tumors decreased significantly with a reduction in NNMT expression (Figure 7c,d). GAP43 expression was upregulated with the downregulation of NNMT expression in solid tumors, which was consistent with the results of cytological experiments. Furthermore, sirtuin 1 (SIRT1) exhibited a decreasing trend (Figure 7e and Figure S5). The results of H&E and Ki-67 staining revealed that tumor proliferation reduced after NNMT knockdown (Figure 7f,g).

### 3.8. Analysis of Differential Genes in Glioma Mice by DNA Methylation Sequencing

Whole-genome bisulfite sequencing (WGBS) [31,34,35] was used to analyze differentially methylated genes (DMGs) on glioma tissues in the two groups of mice. GO analysis of DMGs was also performed to identify candidate genes affecting the acquisition of glioma in mice [36].

**Figure 8:**
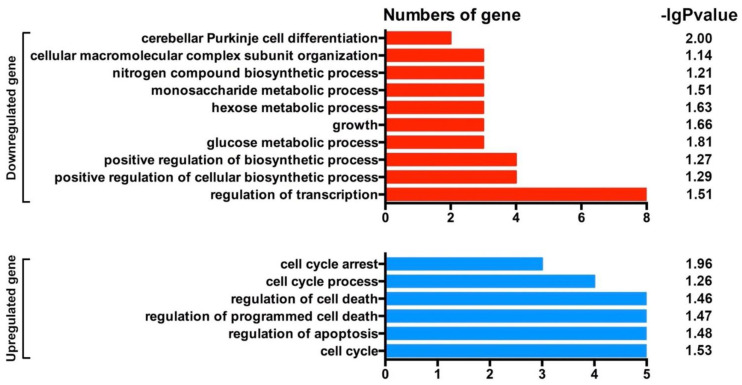
GO analysis of DMGs on glioma in mice. −lg *p* > 1.30 represents significant difference.

DMGs were divided into upregulated and downregulated genes according to their function. GO function enrichment analysis of DMGs was carried out by using the R language cluster profiler package. The upregulated DMGs were significantly enriched in 10 GO entries (*p* < 0.05), including cerebellar Purkinje cell differentiation, cellular macro complex subunit organization, nitrogen compound biological process, monosaccharide metal process, hexose metal process, growth, glucose metal process, positive regulation of biological process, positive regulation of cellular biological process, and regulation of transcription. The downregulated DMGs were significantly enriched in 6 GO entries (*p* < 0.05), including cell cycle arrest and cell cycle process regulation of cell death, regulation of programmed cell death, regulation of apoptosis, and cell cycle. These GO-function-enriched signal pathways revealed that the upregulated signaling pathways were mainly related to tumor growth, and the downregulated signaling pathways were mainly related to tumor growth inhibition (Figure 8).

## 4. Discussion

The diagnosis, treatment, and prognosis of patients with cancer have been critical clinical issues, and a good clinical prognosis largely depends on whether the cancer patients could be treated [37]. The survival of patients with different grades of glioma varies considerably even when the same treatment plan is followed with the same glioma, suggesting that there are limitations in the current clinical diagnosis, treatment, and prognosis of glioma [38].

Fibroblasts create an inflammatory environment that leads to tumor proliferation, and they accumulate more frequently in malignant tissues than in normal tissues. The implication is that fibroblasts could have a key role in malignant tumors. The study of NNMT as a metabolic regulator of cancer-associated fibroblasts is particularly important [21]. In the present study, the potential role of NNMT in glioma progression and prognosis, the associated pathogenic mechanisms, and potential influencing factors were investigated. The results were consistent with those of qRT-PCR and Western blot analyses. Moreover, we observed that upregulation of NNMT expression promoted glioma proliferation and disease progression in patients. Generally, NNMT causes abnormal DNA methylation primarily by acting on the downstream NAD^+^ and methionine metabolic pathways. Cancer cell metabolism requires sustained intracellular production of NAD, and the balance is maintained by the NAD/NADH ratio. In the present study, the results revealed that knockdown of NNMT in low-glucose environments increased the NAD/NADH ratio, with an opposite effect being observed in high-glucose environments. A strong correlation was observed between cancer and diabetes [39], with women with diabetes being 27% more likely to develop cancer than women without diabetes. Similarly, men with diabetes were 19% more likely to develop cancer than those without diabetes [40]. Therefore, we hypothesized that an abnormal increase in the NAD/NADH ratio in glioma leads to abnormal DNA methylation.

GAP43 is a neuron-specific axonal membrane protein, which is involved in neurite cell outgrowth and synapse development, formation, and neuronal cell regeneration, and it can alter cell morphology. GAP43 has been reported to exert inhibitory effects on the proliferation of glioma cells and breast cancer cells, although the associated inhibitory mechanisms remain not known [41,42]. Analysis of the genomic data of colorectal cancer obtained from the TCGA database revealed a substantial silencing of GAP43 gene expression in colorectal cancer, with methylation modification being observed in its promoter region [31,43]. Numerous studies have investigated the role of GAP43 in promoting or inhibiting cancer development [44]. In the current study, the results revealed that NNMT overexpression could compete for DNA methylation, histone methylation, and deacetylation modification processes to epigenetically silence GAP43, thereby inhibiting the oncogenic effect of GAP43, and in turn, tumors become more aggressive and invasive. Sirtuins are an evolutionarily conserved family of NAD^+^-dependent deacetylases and ADP-ribosyltransferases that influence a wide range of biological activities. Among the mammalian sirtuins, SIRT1 is one of the most sought-after members, and it is a regulator of a variety of cellular and organismal processes, including metabolism, immune response, and tumorigenesis [45]. In addition, SIRT1 has a critical function in tissue homeostasis and epigenetic regulation of numerous diseases through deacetylation of histone and nonhistone targets [46]. Activation of SIRT1 in tumor cells can activate AMPK/SIRT1 signaling at higher intracellular NAD^+^ concentrations, and further increase the expression of NF-κB (nuclear factor kappa B) to stimulate Bax activation and cytochrome c release, therefore triggering the cleavage of GSDME by caspase-3, which is a typical pyroptosis protein [47,48].

Several studies regarding NNMT and tumors have been conducted; however, only a few of the studies have reported on NNMT and glioma. In the present study, we analyzed the pathological mechanisms of NNMT in patients with glioma at the clinical, bioinformatic, animal model, and cellular levels. We observed that NNMT promoted SIRT1 and inhibited GAP43 expression, thereby promoting tumor cell proliferation and invasion. Then, through methylation GO analysis, we found that 10 genes could downregulate the methylation process, and 6 genes could upregulate the methylation process, which also confirmed the important role of methylation regulation in the process of glioma invasion and deterioration [49]. The development of NNMT inhibitors is also currently receiving increasing attention, with the basic design principles targeting the structural features of the nicotinamide substrate and elements of the S-adenosyl-L-methionine (SAM) cofactor. The most potent NNMT inhibitor identified so far, 17u, has an IC50 value of 3.7 nM, but the inhibition of tumor growth only appeared when tested at 100 μM concentration in oral, lung, and bladder cancer cell lines, and this difference may be related to the poor cellular permeability of compound 17u [50]. It has been pointed out in the literature that the design of predrug strategies for NNMT inhibitors that can deliver polar NNMT inhibitors into the cells allows NNMT inhibitors to exert stronger efficacy. This provides valuable new insights for the design and optimization of NNMT inhibitors at a later stage [51]. Therefore, NNMT is a key factor influencing the diagnosis, treatment, and prognosis. The results of the present study could be used to inform future clinical treatment decisions or as a basis for future research.

## 5. Conclusions

**Figure 9:**
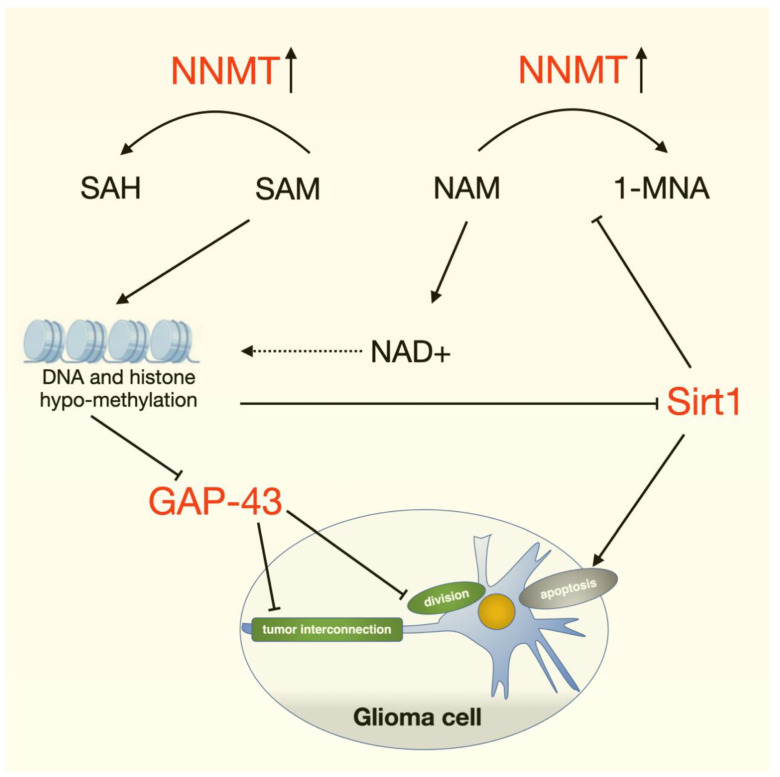
Schematic overview of the role of NNMT in glioma cells. Overexpression of NNMT leads to excessive consumption of SAM and NAM in glioma cells. Methyl donor SAM drain leads to comprehensive DNA/histone demethylation, which affects the expression of a variety of tumor-associated genes such as GAP-43, Sirt1, etc. GAP43 can inhibit the formation of microtubules in glioma cell and intertumor cell network connections. In addition, NAM drain decreases NAD+, a coenzyme of various glucose metabolism enzymes, which is also deeply involved in histone deacetylation. NNMT modulates the NAD-related signaling pathway and downregulates SIRT1, which induces glioma cell apoptosis.

The present study revealed that NNMT is a key factor influencing the occurrence and development of glioma. NNMT knockdown reduced the volume of glioma and improved the prognosis and survival time of patients with glioma. Additionally, overexpression of NNMT epigenetically silenced GAP43 through DNA methylation, histone methylation, and deacetylation modification processes. GAP43 can inhibit the formation of microtubules in tumor and intertumor cell network connections and induce apoptosis through the SIRT1 signaling pathway (Figure 9). Therefore, NNMT could be a potential candidate for the clinical diagnosis and treatment of glioma.

## Figures and Tables

**Table 1:** Primary antibody.

Primary Antibody	Manufacture	Dilution
NNMT	Abcam	1:1000
GAP43	Proteintech	1:1000
Sirt1	CST	1:1000
APOBEC3G	Proteintech	1:1000
CXCR7	Proteintech	1:1000
NEGR1	Abcam	1:1000
PAK3	Abcam	1:1000
TROY	Abcam	1:1000
GAPDH	Proteintech	1:5000
Secondary antibody		
Goat anti-rabbit HRP-IgG	Jackson	1:2000
Goat anti-mouse HRP-IgG	Jackson	1:2000

**Table 2:** Primers.

Sequences	Gene Name
5′-ATATTCTGCCTAGACGGTGTGA-3′	NNMT-F
5′-TCAGTGACGACGATCTCCTTAAA-3′	NNMT-R
5′-AACACAACCGACTACCGAATC-3′	SEMA3F-F
5′-GGCTGCCCAGTGTATAATGAG-3′	SEMA3F-R
5′-CTGTGTCAGAAAAGAGACGGTC-3′	APOBEC3G-F
5′-GTACACGAACTTGCTCCAACA-3′	APOBEC3G-R
5′-TCTGCATCTCTTCGACTACTCA-3′	CXCR7-F
5′-GTAGAGCAGGACGCTTTTGTT-3′	CXCR7-R
5′-AGCCCATCCTTCGAGTACAAA-3′	PAK3-F
5′-TCTTGGTGCGATAACTGGTGG-3′	PAK3-R
5′-GGCCGCAACCAAAATTCAGG-3′	GAP43-F
5′-CGGCAGTAGTGGTGCCTTC-3′	GAP43-R
5′-GGGAGGTGATAAGTGGTCAGT-3′	NEGR1-F
5′-CTGGGTGTATGTTGAGTCTGAAC-3′	NEGR1-R
5′-CCAGCAAGGTCAACCTCGT-3′	TORY-F
5′-CAGAGCCGTTGTACTGAATGT-3′	TORY-R

**Table 3:** Relationship between NNMT expression and the clinical-pathological characteristics of glioma patients from the TCGA GBMLGG cohort data (n = 670).

Characteristic	Levels	Low Expression of NNMT	High Expression of NNMT	*p*
n		335	335	
WHO grade, n (%)	G2	173 (28.2%)	43 (7%)	<0.001
	G3	130 (21.2%)	107 (17.5%)	
	G4	0 (0%)	160 (26.1%)	
IDH status, n (%)	WT	20 (3%)	217 (32.8%)	<0.001
	Mut	314 (47.5%)	110 (16.6%)	
1p/19q codeletion, n (%)	codel	145 (21.8%)	23 (3.5%)	<0.001
	Noncodel	190 (28.6%)	306 (46.1%)	
Primary therapy outcome, n (%)	PD	49 (11%)	54 (12.2%)	<0.001
	SD	96 (21.6%)	48 (10.8%)	
	PR	39 (8.8%)	23 (5.2%)	
	CR	105 (23.6%)	30 (6.8%)	
Gender, n (%)	Female	145 (21.6%)	139 (20.7%)	0.696
	Male	190 (28.4%)	196 (29.3%)	
Race, n (%)	Asian	7 (1.1%)	6 (0.9%)	0.519
	Black or African American	13 (2%)	19 (2.9%)	
	White	311 (47.3%)	302 (45.9%)	
Age, n (%)	≤60	305 (45.5%)	226 (33.7%)	<0.001
	>60	30 (4.5%)	109 (16.3%)	
Histological type, n (%)	Astrocytoma	99 (14.8%)	93 (13.9%)	<0.001
	Glioblastoma	0 (0%)	160 (23.9%)	
	Oligoastrocytoma	87 (13%)	41 (6.1%)	
	Oligodendroglioma	149 (22.2%)	41 (6.1%)	
Age, median (IQR)		39 (32, 50)	54 (40, 63)	<0.001

## Data Availability

The data generated or analyzed during this study are included in this article, or if absent are available from the corresponding author upon reasonable request.

## Supplementary Materials

The following are available online at https://www.mdpi.com/article/10.3390/cancers14143538/s1, Figure S1: The original Western blots of NNMT expression levels of glioma with different grades; Figure S2: The original Western blot of analysis results of the effect with NNMT knock-down. And the original images of proliferation and migration experiment with the glioma cell lines; Figure S3: The original western blots with two different concentrations of Doxycy-cline-induced NNMT knockdown treatment; Figure S4: The original Western blots analysis showed a significant increase in protein expression of NNMT was found after knockdown of the SIRT1 gene in U251 cells; Figure S5: The original images of xenograft tumors isolated from nude mice in the different groups, and the original Western blots of GAP43 and Sirt1 expression. The original images of staining of tumor sections with H&E and Ki-67.
