# Supplementary material for: Overexpression of NNMT in Glioma Aggravates Tumor Cell Progression: An Emerging Therapeutic Target
Source: Cancers (Basel). 2022 Jul 21;14(14):3538. doi: 10.3390/cancers14143538 (PMC9316405; doi:10.3390/cancers14143538)
Supplement: Supplementary file 1 [file cancers-14-03538-s001.zip › cancers-1797023-supplementary.pdf]

The Original Western blots for this article

Figure S1 The original Western blots of NNMT expression levels of glioma with different grades.

NNMT

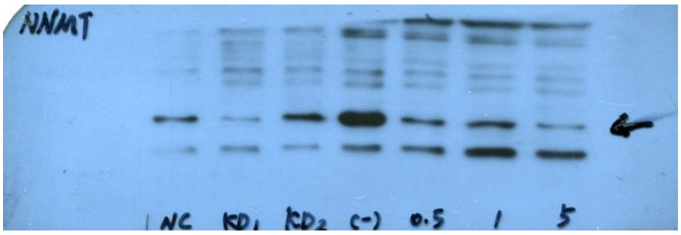

GAPDH

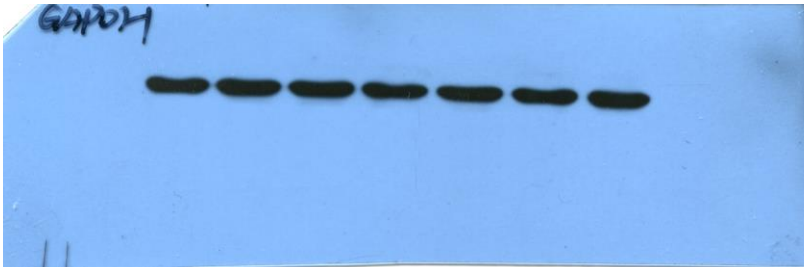

The original Western blots of NNMT expression levels in patients with glioma

Figure S2 The original Western blot of analysis results of the effect with NNMT knockdown. And the original images of proliferation and migration experiment with the glioma cell lines.

Analysis results of the effect of NNMT knockdown as determined by Western blot

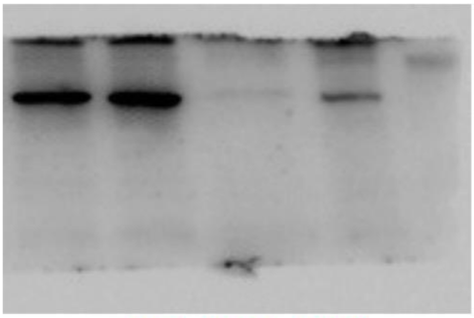

NC KD1 KD2

Figure S3 The original western blots with two different concentrations of Doxycycline-induced NNMT knockdown treatment.

The original western blots with two different concentrations of Doxycycline-induced NNMT knockdown treatment.

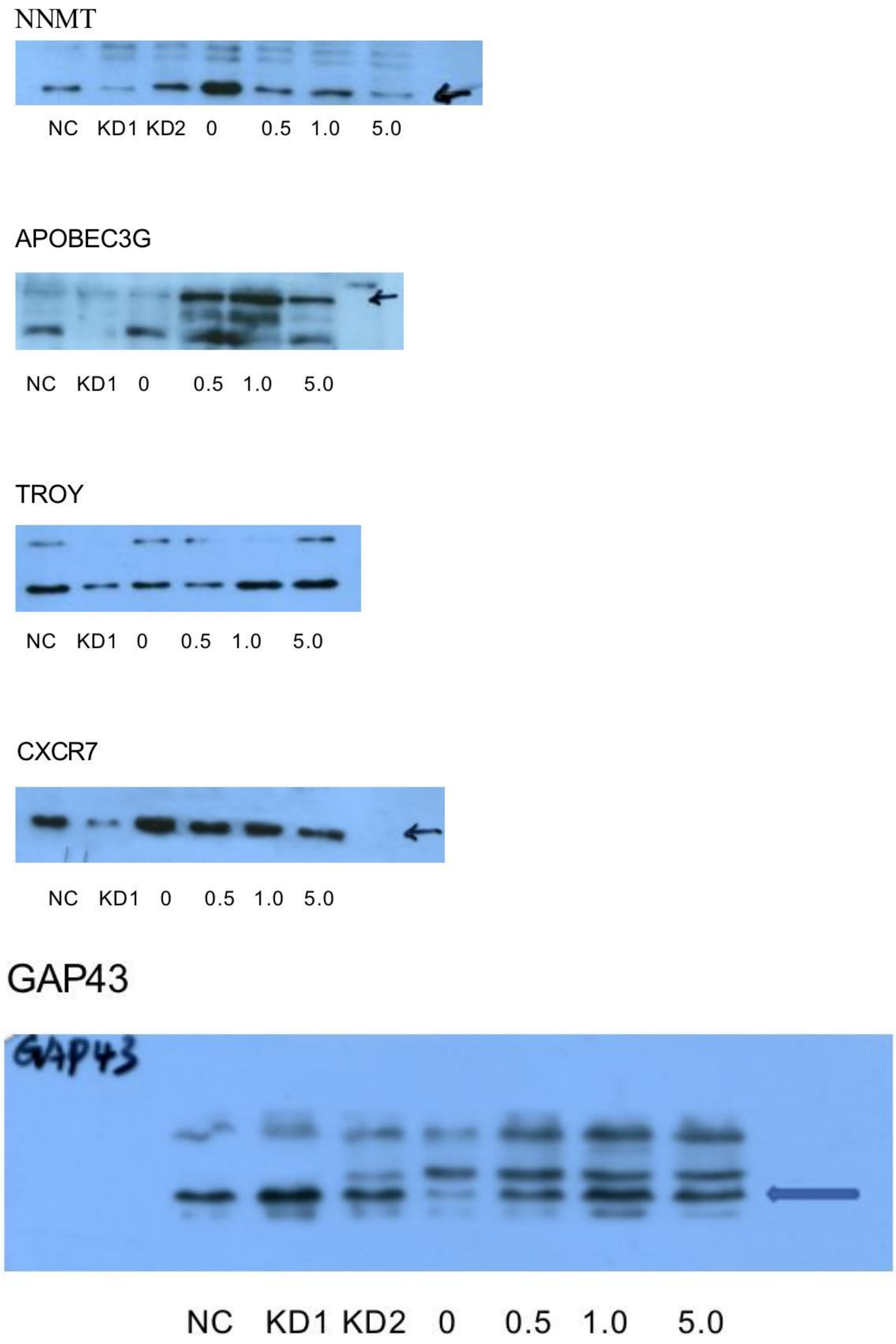

PAK3

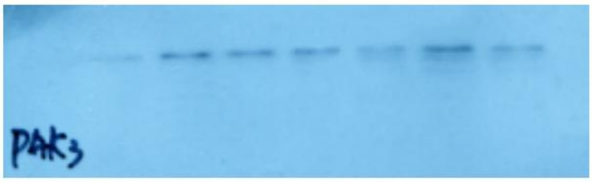

NC KD1 KD2 0 0.5 1.0 5.0

NEGR1

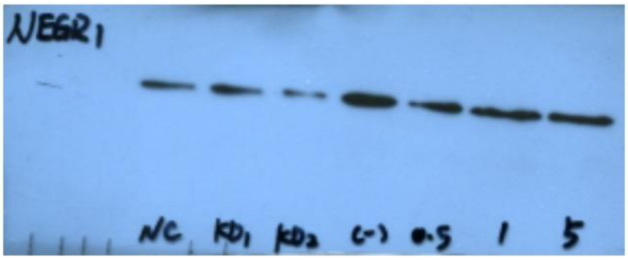

Figure S4 The original Western blots analysis showed a significant increase in protein expression of NNMT was found after knockdown of the SIRT1 gene in U251 cells.

Sirt1

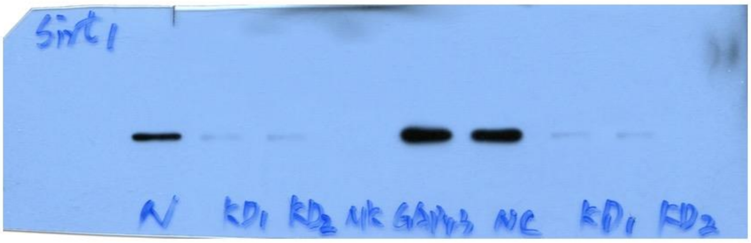

NC KD1 KD2

NNMT

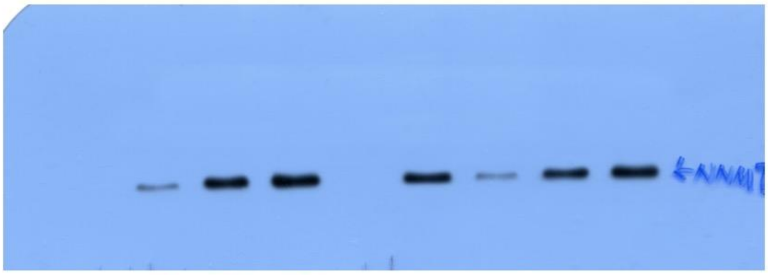

NC KD1 KD2

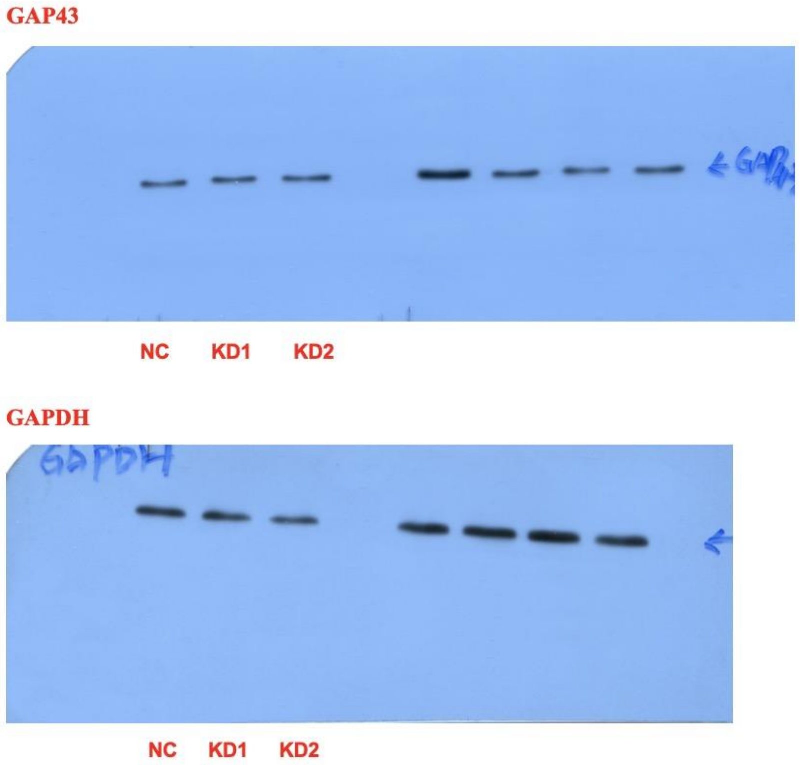

Figure S5 The original images of xenograft tumors isolated from nude mice in the different groups, and the original Western blots of GAP43 and Sirt1 expression. The original images of staining of tumor sections with H&E and Ki-67.

The original Western blots of GAP43 and Sirt 1 expression.

NNMT

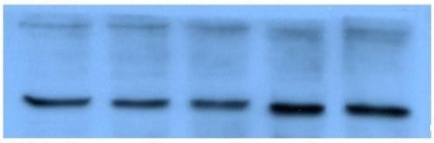

DOX (-) 1 2 3 4 5

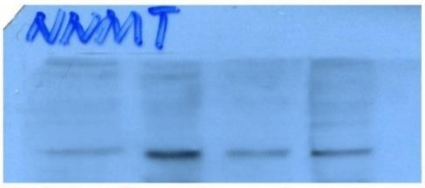

DOX(+) 1 2 3 4

Sirt 1

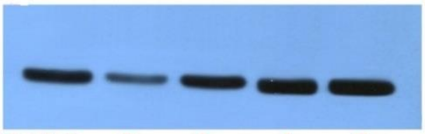

DOX (-) 1 2 3 4 5

The original Western blots of GAP43 and Sirt 1 expression.

NNMT

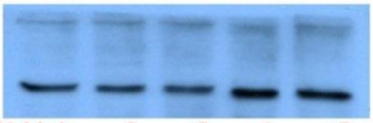

DOX (-) 1 2 3 4 5

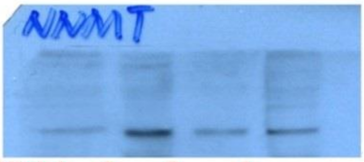

DOX(+) 1 2 3 4

Sirt 1

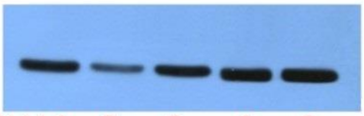

DOX (-) 1 2 3 4 5

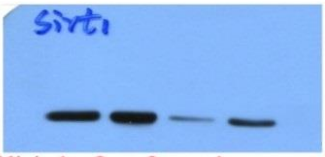

DOX(+) 1 2 3 4

GAP43

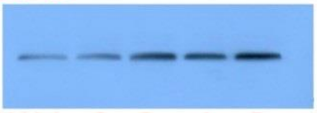

DOX (-) 1 2 3 4 5
